# Basic research on quercetin in liver cancer: Progress and perspectives

**DOI:** 10.1097/MD.0000000000045534

**Published:** 2025-12-12

**Authors:** Binbin Liu, Yunfeng Yu, Ruoyu Wang, Yunan Wu, Kewei Sun

**Affiliations:** a The First Hospital of Hunan University of Chinese Medicine, Changsha, Hunan, China.

**Keywords:** action mechanism, antitumor effect, hepatocellular carcinoma (hcc), liver cancer, quercetin

## Abstract

Quercetin is a natural plant flavonoid that has various biological properties. It is widely found in many fruits, vegetables, and Chinese herbs. Accumulating studies have demonstrated its significant inhibitory effects on liver cancer progression, mainly by inducing tumor cell apoptosis and inhibiting tumor cell proliferation, migration, and invasion. Furthermore, combination therapies incorporating quercetin have been shown to reduce multidrug resistance and potentiate anticancer efficacy. However, the clinical application of quercetin remains challenging due to its poor water solubility and low oral bioavailability. Current research efforts are primarily directed toward developing nano-delivery systems to overcome these pharmacological limitations. Before clinical implementation, systematic safety evaluations must be conducted to ensure therapeutic feasibility. This review comprehensively summarizes recent advances in quercetin-based liver cancer therapeutics, including mechanistic studies, clinical trial developments, nano-delivery system innovations, and safety assessments, and the related mechanisms of quercetin in the field of Traditional Chinese Medicine are summarized, aiming to provide the theoretical basis for the subsequent in-depth mechanism research and clinical development and application.

## 1. Introduction

Hepatocellular carcinoma (HCC), also known as liver cancer, has a relatively high mortality rate. The latest data^[1]^ show that the number of new liver cancer cases worldwide ranks 6th among all malignant tumors every year. The number of deaths from liver cancer is 830,000 per year, ranking 3rd among all malignant tumors.^[1]^ According to the latest estimates of the global burden of liver cancer in 2020, liver cancer cases and deaths are projected to increase by more than 55% by 2040.^[2]^ Based on statistics, there were approximately 431,383 new liver cancer cases and 412,216 new deaths in China in 2022, accounting for approximately half of the global total of new liver cancer patients and deaths.^[3]^ The causes vary, including chronic infection with hepatitis B and C viruses, excessive alcohol consumption, and nonalcoholic steatohepatitis.^[4]^ The treatment of liver cancer includes various traditional methods such as radiotherapy, chemotherapy, surgery, and immunotherapy alone or in combination.^[2,5,6]^ However, due to factors such as the sensitivity of normal cells to radiotherapy,^[7]^ drug resistance to chemotherapy,^[8–10]^ poor liver function, incomplete tumor resection,^[11]^ and the development of endogenous or acquired drug resistance,^[12–14]^ its efficacy and long-term prognosis are unsatisfactory. Therefore, it is imperative to develop new treatment strategies to improve the therapeutic effects in liver cancer as soon as possible. In recent years, plant-derived natural ingredients have provided more options for effective alternatives to conventional chemotherapy drugs.^[15]^

Quercetin is a natural plant flavonoid that exhibits various biological activities. It is found in fruits such as blueberries, apples, and vegetables including onions and broccoli. It is also found in many Chinese herbal medicines, including *Radix bupleuri, Eucommia ulmoides, Peony bark, Artemisia annua*, etc.^[16–19]^ Wu et al^[20,21]^ used a data mining method to screen core Chinese medicines against liver cancer and found quercetin to be an active ingredient. Furthermore, Chinese medicines may exert therapeutic effects on liver cancer through multiple targets and pathways.^[22]^ Quercetin has a rich pharmacological profile with antioxidant, anti-inflammatory, and anticancer properties.^[23–26]^ In the treatment of liver diseases, quercetin significantly affects the occurrence of liver diseases through multiple targets and pathways such as anti-fat accumulation, anti-inflammation, anti-oxidation, and inhibition of apoptosis and proliferation.^[27–29]^ Additionally, in the advanced stage of HCC, quercetin can inhibit cancer cell proliferation, metastasis, and apoptosis through relevant signaling pathways.^[30]^ Quercetin is effective and has very low acute toxicity, and no toxicity at low doses,^[31]^ making it safer and more effective than the side effects produced by radiotherapy, chemotherapy, interventions, and other therapeutic modalities.^[32]^ In recent years, many studies have been conducted on the use of quercetin for the treatment of liver cancer. This article reviews the mechanism of quercetin in treating liver cancer through the regulation of cell proliferation, apoptosis, and migration and provides a reference for the research and development of the anti-liver cancer effect of quercetin and its clinical application. Table 1 reviews the anti-liver cancer activities of quercetin.

**Table 1:** Possible mechanisms, real modules, targets, doses and reference of quercetin in liver cancer.

Possible mechanisms	Real modules (animal/ cell)	Targets	Doses	References
Proliferation	KIM-1, KYN-1, KYN-2, KYN-3, HAK-1A, HAK-1B, HAK-2, HAK-3, HAK-4, HAK-5, HAK-6, KMCH-1 and KMCH-2	Cell cycle arrest	0, 12.5, 25, 50, and 100 μM	Hisaka et al^[33]^
	HepG2.2.15, Hep3B	CDK1	323.29 μM, 165.81 μM	Li et al^[34]^
	LM3	IL-6, JAK2, STAT3, PCNA	80, 20 μmol/L	Wu et al^[35]^
	HepG2	ROS, Cyclin A, CHK1, HO-1	20, 40, 80, and 100 μM	Jeon et al^[36]^
	SNU-449, Hep3B	Nosip	50 and 100 μM, 150 and 200 μM	Gao et al^[37]^
	HepG2, SNU-449	PI3K, Akt, GLUT4, IRS-2	50μM	Tu et al^[38]^
Apoptosis	LM3	JAK2, STAT3, Bax	80, 20 μmol/L	Wu et al^[35]^
	adult male Sprague Dawley rats	CK2α, Caspase-3, Caspase-8	100 mg/kg	Salama et al^[39]^
	SMMC7721, HepG2, Male BALB/c nude mice	Akt, mTOR, MAPK, Bax, Bcl-2, caspase-3	21 μM, 34 Μm, 60 mg/kg	Ji et al^[40]^
	HepG2	Akt, Bcl-2, SK1	80 μM	Momchilova et al^[41]^
	HepG2	p53, YY1, Bax, Bcl-2	10, 20, and 30 µM	Guan et al^[42]^
	Adult male Wistar rats	p53, Bcl-2	10 mg/kg	Ahmed et al^[43]^
Metastasis and invasion	LM3	IL-6, JAK2, STAT3, N-cadherin, E-cadherin, MMP9, vimentin	80, 20 μmol/L	Wu et al^[35]^
	HepG2; HuH-6	FZD4, SIRT6, Wnt5a, β-catenin	120 μM	Liu^[44]^
	HuH7	HGF, TGF-α, Akt	7, 30 μM	Yamada et al^[45]^

Bax = Bcl-2-associated X protein, Bcl-2 = B-cell lymphoma-2, CDK1 = cyclin-dependent kinase 1, FZD4 = frizzled 4, GLUT4 = glucose transporter 4, HGF = hepatocyte growth factor, IL-6 = interleukin-6, IRS-2 = insulin receptor substrate 2, MAPK = mitogen-activated protein kinase, mTOR = mammalian target of the rapamycin, Nosip = nitric oxide synthase-interacting protein, SK1 = sphingosine kinase 1, TGF-α = transforming growth factor-α.

## 2. Retrieval strategy and method

This review was collected by searching PubMed, CNKI databases, and www.clinicaltrials.gov related to quercetin treatment of HCC. The literature search was conducted within the last 5 years up to November 11, 2024.

Search terms included but were not limited to “Quercetin,” “Cancer of Liver,” “Hepatocellular Cancer,” “Hepatocellular Carcinoma,” “Hepatic Cancer,” “Liver Cancer,” and “Clinical Trial.” Boolean operators (AND/OR) were used to efficiently combine these terms and ensure that the search was both comprehensive and accurate.

The inclusion criteria for the studies were as follows: Investigate studies on various mechanisms of quercetin in the treatment of HCC. Report studies on the bioavailability and pharmacokinetics of quercetin. Focus on studies on quercetin in combination with other drugs in the treatment of HCC. Report studies on the nano-delivery of quercetin in HCC. Focus on studies related to the safety of quercetin. Report studies on the use of quercetin-containing single Chinese herbal medicines or Chinese herbal medicine combinations in the treatment of related studies in liver cancer. Studies were excluded if they were not directly related to these areas or were not in English. The search was not limited by study design and included a wide range of in vivo and in vitro experiments, and clinical trials. The search was initially screened based on title and abstract, and if the inclusion criteria were met, the full text was obtained for further assessment of relevance.

## 3. Chemical properties of quercetin

**Figure 1.**
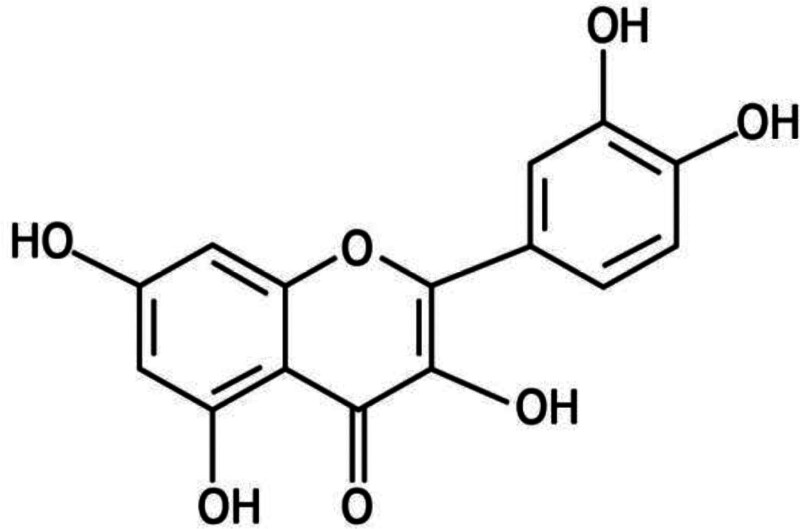
Chemical structure of quercetin.

The structure of quercetin is 2-(3,4-dihydroxy phenyl)-3,5,7-trihydroxy-4H- chrome-4-one with a melting point of 316°C and a molecular formula of C_15_H_10_O_7_.^[46]^ Its chemical name is 3, 3′, 4′, 5, 7 pentahydroxyflavone.^[47]^ Quercetin is found in plants mainly in the form of glycosides.^[48]^ The bioavailability of quercetin is significantly limited by its extensive intestinal and first-pass metabolism.^[49]^ Gut microbiota (GM)-derived β-glucosidase catalyzes the hydrolysis of the quercetin glycosidic bond to produce a quercetin aglycone. Quercetin aglycone is subsequently absorbed into enterocytes and metabolized to quercetin couplings that enter the blood and lymphatic circulation.^[50]^ Oral quercetin is excreted via urine and feces, whereas some quercetin metabolites are excreted via the biliary tract.^[51]^ Quercetin is highly lipophilic by nature owing to the presence of 5 hydroxyl groups (Fig. 1), resulting in very poor aqueous solubility (approximately 1 μg/mL) and relatively low bioavailability (0.17–7 μg/mL).^[52]^ To improve its utilization, researchers have begun to develop various techniques that allow quercetin to be encapsulated in another material to exert pharmacological effects.^[53,54]^

## 4. Study on the mechanism of quercetin against liver cancer

### 4.1. Inhibition of liver cancer cell proliferation

Uncontrolled cell proliferation is a characteristic of tumor cells. Unrestricted cell proliferation due to cell cycle dysregulation is an important factor in tumor development.^[55]^ To meet their rapid growth requirements, cancer cells undergo metabolic adaptations to survive under multiple stressful conditions such as hypoxia and nutrient deprivation.^[56]^

#### 4.1.1. Promotes cell cycle arrest

Hisaka et al study^[33]^ focused on 11 HCC cell lines and 2 combined human HCC and cholangiocarcinoma cell lines. Through MTT assay, quercetin was found to inhibit the proliferation of HCC cells by inducing apoptosis and cell cycle arrest. Cyclin-dependent kinase 1 (CDK1), a key cell cycle protein, is involved in HBV-associated HCC.^[57]^ In normal cells, the expression of CDK1 is restrictively regulated, however, in cancer, its activity is enhanced in various ways.^[58]^ Therefore, inhibition of CDK1 expression can be considered as a strategy to suppress the proliferation of tumor cells. Li et al^[34]^ used bioinformatics to predict CDK1 as a potential target of HBV-associated HCC, and then searched for the treatment of HBV-associated HCC by reverse network pharmacological analysis of the major herbal small molecules, quercetin being one of them. The cell counting kit-8 assay further revealed that quercetin exerted a concentration-dependent inhibitory effect on the proliferation of HepG2.2.15 and Hep3B cells, and reduced the expression of CDK1, one of the predicted key targets.

#### 4.1.2. Inhibition of Janus kinase 2/signal transducer and activator of transcription 3 (JAK2/STAT3) signaling pathway

Levels of interleukin-6 (IL-6), phosphorylated JAK2, and phosphorylated STAT3 were significantly increased in HCC cells. Treatment with JAK2 inhibitors and IL-6 neutralizing antibodies significantly inhibits the proliferation rate of HCC cells.^[59]^ IL-6 binds to membrane receptors and induces activates the downstream JAK2/STAT3 pathway. This pathway has been shown to play an important role in cancer cachexia and is significantly associated with the proliferation, invasion, and migration of cancer cells.^[60,61]^ IL-6, a major inflammatory mediator in the tumor microenvironment (TME), acts directly on tumor cells to induce the expression of STAT3 target genes, which encode proteins that drive tumor proliferation.^[62]^ Wu et al^[35]^ found through the cell counting kit-8 test that quercetin inhibits HCC cell proliferation in a time- and dose-dependent manner, which is related to the IL-6/JAK2/STAT3 signaling pathway. Quercetin-induced HCC cells were blocked in the S and G2/M phases and the expression of Bcl-2-associated X protein (Bax) was upregulated. The results of this study suggested that the apoptosis-inducing effect of quercetin may depend on its regulation of the cell cycle.

#### 4.1.3. Down-regulation of reactive oxygen species (ROS) levels

Hyperproliferation of cancer cells requires the maintenance of high intracellular ROS.^[63]^ Therefore, down-regulation of intracellular ROS levels in cancer cells inhibits cell proliferation, leading to apoptosis.^[64,65]^ The transcriptional regulator p53 is an oncogenic protein that inhibits cancer cell proliferation by inducing cell cycle arrest or apoptosis.^[66]^ It was found that inhibition of HepG2 cell proliferation by quercetin might be associated with down-regulation of intracellular ROS levels and up-regulation of p53.^[36]^ Further findings suggested that the intracellular ROS level was one of the factors determining the anticancer effect of quercetin, and was not related to the expression of p53. Another study found that quercetin also affected ROS metabolism by effectively reducing the pool of glutathione at intracellular levels.^[67]^

#### 4.1.4. Inhibition of nitric oxide synthase-interacting protein (Nosip) expression

Nosip interacts with nitric oxide (NO) synthase to regulate the synthesis and release of NO.^[68]^ Heinrich^[69]^ found that NO has both pro-tumorigenic and tumor-suppressor effects, with high concentrations inhibiting tumorigenesis and low concentrations having tumor-promoting effects. One study^[37]^ found that overexpression of Nosip promotes HCC cell proliferation, where low expression of Nosip inhibits the proliferation of HCC cells. Meanwhile, this study confirmed that quercetin could inhibit the proliferation of HCC cells using in vitro cellular experiments, and found that quercetin caused a decrease in the expression of Nosip after acting on HCC cells. Based on these results, they proposed that quercetin might inhibit the proliferation and motility of HCC cells by suppressing the expression of Nosip.

#### 4.1.5. Inhibition of phosphoinositide 3-kinases/protein kinase B (PI3K/Akt) signaling pathway

**Figure 2.**
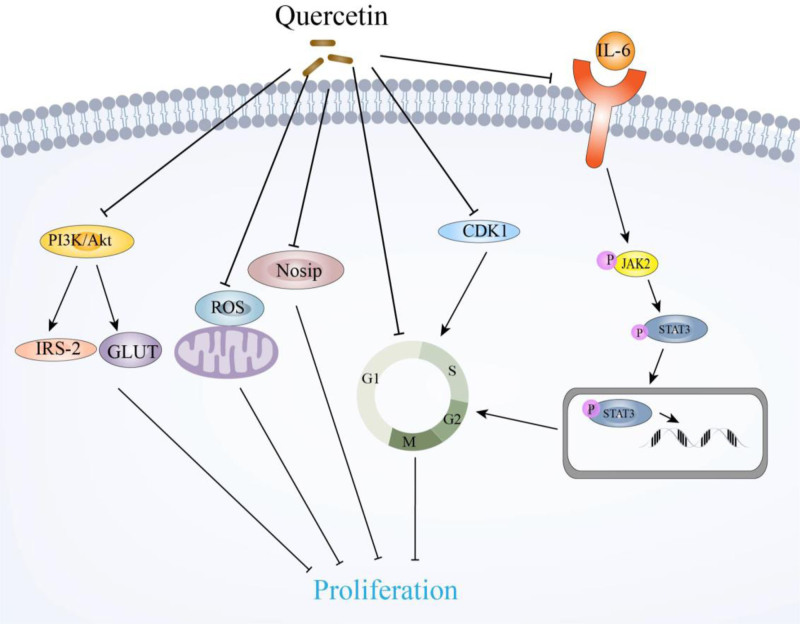
Mechanisms of quercetin suppressing proliferation in liver cancer. This figure summarizes the main mechanisms by which quercetin inhibits the proliferation of HCC cells. It can be seen that quercetin inhibits cell proliferation mainly by promoting cell cycle arrest, inhibiting the JAK2/STAT3 and PI3K/Akt signaling pathway, and down-regulating ROS levels. Akt = protein kinase B, CDK1 = cyclin-dependent kinase 1, GLUT = glucose transporter 4, HCC = hepatocellular carcinoma, IL-6 = interleukin-6, IRS-2 = insulin receptor substrates 2, JAK2 = Janus kinase 2, Nosip = nitric oxide synthase-interacting protein, PI3K = phosphoinositide 3-kinases, ROS = reactive oxygen species, STAT3 = signal transducer and activator of transcription 3.

The PI3K/Akt pathway is essential for cell proliferation and apoptosis and is closely related to tumor growth.^[70]^ Tu^[38]^ found that quercetin effectively inhibits the proliferation of HCC cells and induce apoptosis. Further experiments showed that quercetin inhibited the mRNA levels of PI3K and Akt and the downstream genes of the PI3K/Akt pathway, glucose transporter 4, and insulin receptor substrate 2, in HCC cells. This suggests that quercetin inhibits the malignancy of liver cancer cells through the PI3K/Akt signaling pathway. Inhibitors targeting the PI3K/Akt signaling axis and different downstream pathways in HCC are currently undergoing clinical trials.^[71]^ Figure 2 shows the various mechanisms by which quercetin suppresses the proliferation of liver cancer cells.

### 4.2. Induction of liver cancer cell apoptosis

Apoptosis is a process of programmed cell death, which is a natural way of removing aging cells in vivo.^[72]^ In cancer cells, evasion of apoptosis is crucial for maintaining sustained proliferation and tumor formation. The ability to inhibit apoptosis is regarded as one of the hallmarks of cancer.^[73]^ In addition, dysregulation of apoptosis is a key mechanism contributing to drug resistance and the progression of liver cancer.^[74–76]^

#### 4.2.1. Inhibition of CK2α expression

CK2α, a subtype of the catalytic subunit of the protein serine/threonine kinase CK2, is overexpressed in liver cancer. Inhibition of CK2α expression can significantly promote apoptosis in HCC cell lines, and the mechanism is related to the expression of apoptosis-related proteins.^[77]^ Salama et al^[39]^ found that quercetin, in addition to its antioxidant activity, can also inhibit the expression of CK2α and induce apoptosis in liver cancer cells. Caspase-8 is an initiator of the exogenous apoptotic cascade,^[78]^ and the inhibition of caspase-8 activity has been reported in HCC.^[79]^ In Salama study quercetin reduced caspase-8 mRNA levels in both protected and treated groups, suggesting that quercetin may not be directly involved in the exogenous apoptotic pathway.^[39]^

#### 4.2.2. Induction of autophagy

Autophagy, a type II programmed cell death process, has been shown to co-regulate cell death via apoptosis.^[80]^ Increasing evidence has confirmed that autophagy can also induce cell death.^[81]^ Ji et al^[40]^ found that quercetin induced apoptosis by promoting autophagy through the inhibition of the Akt/mammalian target of the rapamycin (mTOR) pathway and activating the mitogen-activated protein kinase (MAPK) pathway through in vivo and in vitro experiments. Thus, inhibition of autophagy significantly attenuates the effects of quercetin on tumor growth inhibition and apoptosis induction.^[40]^ These results suggest that autophagy may be an important mechanism for quercetin-induced apoptosis in cancer cells. Relevant studies have shown that the Akt/mTOR signaling pathway is a classical and important negative regulatory pathway for autophagy^[82]^ that the MAPK signaling pathway is involved in cancer development and progression through the regulation of cell apoptosis and autophagy.^[83]^

#### 4.2.3. Alteration of sphingolipid metabolism

Sphingolipids can affect cancer cell death and proliferation and are also potential chemotherapeutic agents.^[84]^ Sphingosine kinase 1 is one of the most important sphingolipid metabolizing enzymes that affect apoptosis and metastatic capacity.^[85]^ Momchilova et al^[41]^ studied the influence of 2 sphingolipid-related agents, quercetin and fingolimod, on the sphingolipid metabolism in HepG2 cells. These results imply that combined treatment can result in a more significant downregulation of sphingosine kinase 1 activity and protein expression. The results also showed that quercetin alone or in combination with fingolimod reduced Akt phosphorylation and B-cell lymphoma-2 (Bcl-2) expression, leading to increased apoptosis in HepG2 cells. Activated Akt inhibits the expression of pro-apoptotic proteins while up-regulating the expression of antiapoptotic proteins.^[86]^ Decreasing Akt phosphorylation and decreasing the expression of Bcl-2 can promote apoptosis.

#### 4.2.4. Upregulation of tumor suppressor p53 expression

**Figure 3.**
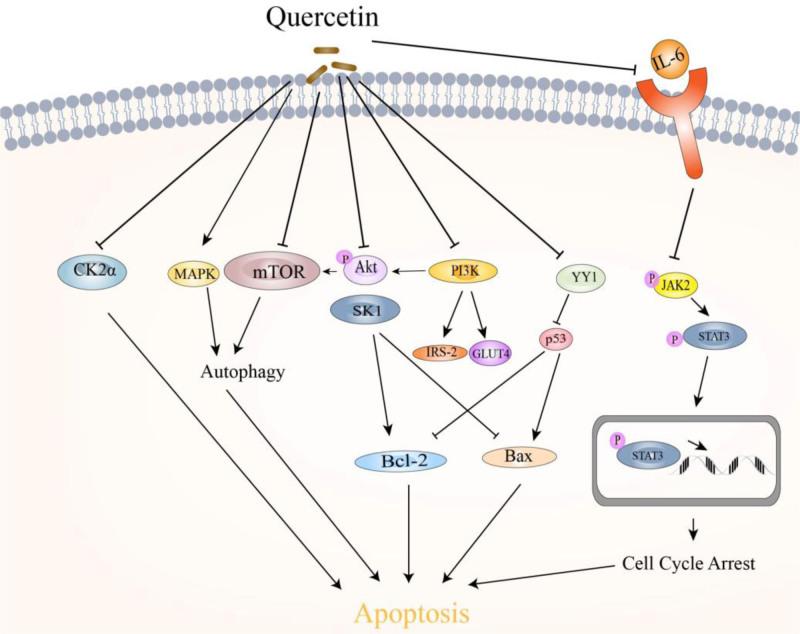
Mechanisms of quercetin inducing apoptosis in liver cancer. This figure summarizes the main mechanisms by which quercetin induces the apoptosis of HCC cells. It can be seen that quercetin mainly affects the apoptosis of HCC cells by inhibiting the expression of CK2α, promoting autophagy, altering sphingolipid metabolism, up-regulating the expression of p53, and inhibiting the PI3K/Akt and JAK2/STAT3 signaling pathways. Akt = protein kinase B, Bax = Bcl-2-associated X protein, Bcl-2 = B-cell lymphoma-2, CK2α = CK2 alpha, GLUT = glucose transporter 4, HCC = hepatocellular carcinoma, IL-6 = Interleukin-6, IRS-2 = insulin receptor substrates 2, JAK2 = Janus kinase 2, MAPK = mitogen-activated protein kinase, mTOR = mammalian target of rapamycin, p53 = tumor suppressor p53, PI3K = phosphoinositide 3-kinases, SK1 = sphingosine kinase 1, STAT3 = signal transducer and activator of transcription 3, YY1 = Yin Yang 1.

As a tumor suppressor, the transcriptional regulatory factor p53 can induce apoptosis and inhibit tumor development by regulating the expression of factors such as the pro-apoptotic protein Bax and antiapoptotic protein Bcl-2.^[87]^ Guan et al^[42]^ proposed that quercetin competes with p53 for the binding site of Yin Yang 1 (YY1) by binding to the multifunctional transcription factor YY1 protein, thereby disrupting the interaction of YY1-p53 and promoting the activation of p53. Furthermore, it promoted the downstream expression of Bax and inhibited the expression of Bcl-2, thus inducing apoptosis in HepG2 cells. When studying the effect of quercetin on liver cancer induced by diethylnitrosamine/2-acetylaminofluorene, Ahmed^[43]^ found that quercetin treatment could up-regulate the expression of p53 to enhance cell apoptosis. Figure 3 shows various mechanisms of quercetin-induced apoptosis in liver cancer cells.

### 4.3. Inhibition of liver cancer cell invasion and migration

Cancer metastasis is the spread of cancer cells from the primary lesion to distant organs and is considered the main cause of cancer-related deaths.^[88]^ The invasiveness of tumor cells is an important factor in the malignant development of tumors, and inhibition of tumor metastasis and invasion is one of the cancer treatment methods.^[89]^

#### 4.3.1. Inhibition of the JAK2/STAT3 signaling pathway

Epithelial-mesenchymal transition (EMT) is the process by which epithelial cells acquire a mesenchymal stem cell phenotype. Cells lose intercellular and intercellular matrix adhesion and acquire a mesenchymal cell phenotype, which allows them to separate from the primary tumor and isolate surrounding tissues and distant organs.^[90]^ It has been demonstrated that activation is a key process in cancer cell metastasis.^[91]^ Wu et al^[35]^ found that quercetin could modulate the expression levels of EMT biomarkers in the HCC cell line LM3, up-regulating the expression of E-cadherin and down-regulating the expression of N-cadherin and vimentin, thereby reversing the EMT process. Quercetin also inhibits the expression of matrix metalloproteinase 9. Studies have shown that matrix metalloproteinase 9 plays an important role in human cancer invasion and metastasis.^[92]^ These processes are ultimately related to the JAK2/STAT3 signaling pathway.

#### 4.3.2. Inhibition of the wingless-related integration (Wnt)/β-catenin signaling pathway

Sirtuins (SIRT) are NAD^+^-dependent lysine deacetylases that play important roles in various cellular processes and biochemical activities.^[93]^ As one of the NAD-dependent deacetylases, SIRT6 was found to play a role by promoting HCC cell invasion, migration, and EMT as an oncogene in HCC.^[94]^ As a number of unconventional G protein-coupled receptor families, frizzled 4 was reported to activate diverse signaling pathways, including Wnt/β-catenin signaling.^[95]^ Liu^[44]^ found that quercetin inhibited HepG2 cell invasion by upregulating the expression of SIRT6 to reduce frizzled 4 expression and inhibit the Wnt/β-catenin pathway. Numerous pieces of evidence suggest that the Wnt/β-catenin pathway is closely related to tumor invasion and migration.^[96–98]^

#### 4.3.3. Inhibition of Akt signaling pathway

**Figure 4.**
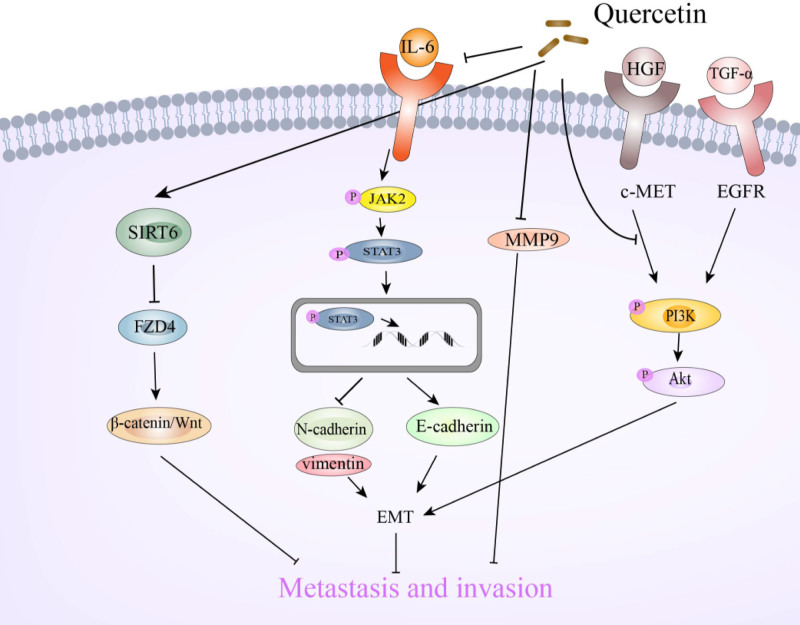
Mechanisms of quercetin suppressing metastasis and invasion in liver cancer. This figure summarizes the main mechanisms by which quercetin inhibits the metastasis and invasion of HCC cells. It can be seen that quercetin affects cell metastasis and invasion mainly by inhibiting the Wnt/β-catenin, JAK2/STAT3, and Akt signaling. Akt = protein kinase B, c-MET = c-mesenchymal-epithelial transition factor receptor, EGFR = epidermal growth factor receptor, EMT = epithelial-mesenchymal transition, FZD4 = frizzled 4, HCC = hepatocellular carcinoma, HGF = hepatocyte growth factor, IL-6 = interleukin-6, JAK2 = Janus kinase 2, MMP9 = matrix metallopeptidase 9, PI3K = phosphoinositide 3-kinases, SIRT6 = sirtuin 6, STAT3 = signal transducer and activator of transcription 3, TGF-α = transforming growth factor-α, Wnt = wingless-related integration.

Studies have reported the antitumor activity of quercetin through the Akt pathway, including in HCC.^[40]^ Both hepatocyte growth factor (HGF) and transforming growth factor-α (TGF-α) are involved in HCC cell invasion and metastasis, respectively.^[99,100]^ Previous studies have also demonstrated that HGF and TGF-α induce the migration of human HuH7 cells and that the p38 MAPK and Akt signaling pathways are involved in the migration of cells induced by these growth factors.^[101–104]^ Yamada et al^[45]^ suggested that quercetin significantly attenuates the phosphorylation of PI3K and inhibits HGF- and TGF-α-induced Akt activation. They found that quercetin inhibited growth factor-induced migration through the PI3K/Akt signaling pathway but not the p38 MAPK signaling pathway, which may provide new insights. Regarding the downstream of the PI3K/Akt pathway, it has been reported that quercetin can inhibit cancer aggressiveness by reversing EMT through downregulation of epidermal growth factor receptor and its downstream PI3K/Akt pathway.^[105]^ Figure 4 shows the various mechanisms by which quercetin suppresses the metastasis and invasion of liver cancer cells.

### 4.4. Reducing resistance to chemotherapy in liver cancer

Chemotherapy is one of the most effective strategies for liver cancer treatment. Patients tend to be sensitive to chemotherapy initially, and after a period of treatment, most develop acquired resistance. Studies have shown that metastasis and drug resistance are major contributors to tumor treatment.^[106]^

#### 4.4.1. Quercetin combined with sorafenib (SFB) reduces drug resistance

SFB, a first-line targeted drug for advanced liver cancer treatment, is a multityrosine kinase inhibitor that inhibits cancer cell angiogenesis and proliferation.^[107]^ Abdelmoneem et al^[108]^ studied the co-delivery of quercetin preformulated with SFB as a phospholipid complex via protein-shell-oil-core nanocapsules (NCs). Inspired by the targeting effect of lactoferrin (LF) by binding to the LF receptor overexpressed by HCC cells, they electrostatically deposited LF shells onto drug-carrying oily nuclei to prepare LF shell-oil nuclei NCs. In this study, it was found that dual-targeted LF-NCs co-delivering SFB and quercetin exhibited potent in vivo antitumor efficacy and could be used as a potential therapeutic strategy for HCC. Abdu et al^[109]^ demonstrated through in vitro and in vivo experimental results that quercetin alone or in combination with SFB, significantly inhibited HCC growth, induced cell cycle arrest, and induced apoptosis and necrosis. Further molecular data have indicated that quercetin alone or in combination with SFB down regulates key inflammation-, proliferation-, and angiogenesis-related genes tumor necrosis factor-alpha (TNF-α), vascular endothelial growth factor, p53, and nuclear factor kappa B (NF-κB). A recent study^[110]^ reported that quercetin significantly inhibits proliferation, stimulates apoptosis, and enhances the efficacy of SFB in the treatment of Huh7(R). The potential mechanisms may be related to epidermal growth factor receptor tyrosine kinase inhibitor resistance and the PI3K-Akt signaling pathway.

#### 4.4.2. Quercetin combined with gemcitabine (GEM) reduces drug resistance

GEM is a chemotherapeutic drug commonly used to treat advanced liver cancer.^[111]^ GEM is a deoxycytidine nucleoside analog that exerts anticancer effects by directly interfering with DNA synthesis, inhibiting ribonucleotide reductase, and blocking G1/S phase cell cycle progression.^[112]^ Drug resistance is the most common reason for the failure of GEM chemotherapy. Through proliferation experiments, Liu et al^[113]^ found that quercetin had cytotoxic effects on GEM-resistant cell lines (HepG2 and PANC-1), and flow cytometry analysis showed that quercetin had significant pro-apoptotic effects. Compared to GEM alone, treatment with quercetin combined with GEM increased the antitumor effect. Quercetin caused S-phase blockade in GEM-resistant cell lines, and western blot analysis showed upregulation of tumor protein p53 and downregulation of cell cycle protein D1 (cyclin D1). This study suggests that quercetin adjuvant therapy may reduce drug resistance in GEM therapy, thus enhancing the antitumor effect and providing a new idea for the mechanistic study of quercetin therapy for HCC. Hypoxia-inducible factor expression is associated with chemotherapy resistance.^[114]^ Hassan et al^[115]^ found that the combination of GEM and doxorubicin with quercetin down-regulated the expression of hypoxia-inducible factor and increased the expression of the apoptosis-regulating factor p53, thus promoting apoptosis and reducing chemotherapy resistance.

## 5. Other drug combinations

For cancer patients, single-use drugs are often prone to induce drug resistance. Therefore, in most cases, drug combinations can have a synergistic effect and enhance anticancer treatment. Table 2 summarizes the studies of quercetin in combination with other drugs in treating liver cancer.

**Table 2:** Drug combination (includes combinations that reduce resistance in combination with chemotherapy drugs), real modules, possible mechanisms, targets, doses and reference of quercetin in liver cancer.

Drug combination	Real modules (animal/ cell)	Possible mechanisms	Targets	Doses	References
Quercetin+Sorafenib	HepG2; HCC-bearing mice	Apoptosis and antiangiogenic effect, proliferation	NF-κB, TNF-α, VEGF, caspase-3 and Ki-67	0–100 µM + 0–100 µM;10 mg/kg + 10 mg/kg	Abdelmoneem et al^[108]^
Quercetin+Sorafenib	HepG2; wistar albino-strain adult male rats	Cell cycle arrest, apoptosis, necrosis, oxidative stress and inflammation	TNF-a, VEGF, P53, NF-kB, ALT, AST, GSH, AFP, PIVKA-II, CRP, IL-6 and LDH	10.9 µM + 107.7 µM; 50 mg/kg + 7.5 mg/kg	Abdu et al^[109]^
Quercetin+Sorafenib	Huh7; SPF grade BALB/c female nude mice	Apoptosis and proliferation	EGFR, p-Akt/Akt, p-ERK/ERK, Bcl-2, Cleaved PARP1 and Bax	64 μM + 16 μM;50 mg/kg/d + 30 mg/kg/d	Zhang et al^[110]^
Quercetin+Gemcitabine	HepG2 and Huh7	Apoptosis, proliferation and cell cycle arrest	p53 and cyclin D1	200 mM + 38 mg/mL	Liu et al^[113]^
Quercetin+Gemcitabine; Quercetin+Doxorubicin	HepG2	Apoptosis	p53, cleaved caspase-3 and HIF-1α	50 μM + 5 μg/mL; 50 μM + 10 μM	Hassan et al^[115]^
Quercetin+Balsamin	HepG2	Apoptosis	Caspase-3 and caspase -8	37 Μm + 25 μg/mL	Ajji et al^[116]^
Quercetin+SeNPs	Adult male Sprague–Dawley rats	Oxidative stress, fibrosis and inflammation	p53, β‐catenin, cyclin D1 and IL‐33	10 mg/kg + 5 mg/kg	Mohamed et al^[117]^
Quercetin+Arsenic trioxide + Aloe-emodin	HepG2	Proliferation and apoptosis	Bax, annexin V, Bcl-2 and hTERT	60 µM + 4 µM + 60 µM	Chaudhary et al^[118]^
Quercetin+anti-programmed cell death 1 (anti-PD-1) antibody	C57L/J mice	Gut microbiota, macrophage immunity and tumor microenvironment	CD8a, CD4, CD11b, IL-4, IL-6, IL-10, IFN-γ, GMCSF, G-CSF and TLR4, p-IκBα, p65, PD-L1	100 mg/kg·d + 20 mg/kg (twice a week)	Wu et al^[119]^

Bax = Bcl-2-associated X protein, Bcl-2 = B-cell lymphoma-2, IL-6 = interleukin-6, NF-κB = nuclear factor kappa B, TNF-α = tumor necrosis factor-alpha, VEGF = vascular endothelial growth factor.

Ajji^[116]^ found that balsamin combined with flavonoids (quercetin, naringenin, and naringin) reduced the viability of HepG2 cells and induced apoptosis by activating caspase-3 and caspase-8, up-regulating pro-apoptotic genes(Bax and p53), and down-regulating of antiapoptotic genes (Bcl-2 and Bcl-XL). The combination increased mitochondria-mediated apoptosis with a possible additive effect in HepG2 cells compared to its use alone. Future studies should focus on evaluating the efficacy and appropriate dosage of the combination in clinical trials.

Selenium (Se) is an essential trace element required for human health. Se deficiency has been associated with various diseases and many types of cancers, including HCC.^[120]^ Meanwhile, researchers found that Se nanoparticles were able to overcome SFB resistance by modulating apoptosis and mTOR/NF-κB signaling in a rat model of HCC.^[121]^ Mohamed et al^[117]^ suggested that the combination of quercetin and Se nanoparticles resulted in a higher return to normal levels of AFP and more normal histopathological features than compared to the drug alone. There was also an improvement in liver function parameters and a decrease in the percentage of fibrosis and necroinflammation scores compared to those in the HCC group. Further studies have shown that quercetin, in combination with Se nanoparticles, could be effective in overcoming resistance to SFB by modulating oxidative stress, inflammatory state, and the p53/β-catenin/cyclin D oncogenic axis pathway to inhibit HCC progression in rats.^[117]^ Wnt/β-catenin signaling governs vital embryonic and somatic processes. This pathway is initiated through the combination of Wnt and its receptor Frizzled, stimulating the accumulation of β-catenin in the cytoplasm and translocation to the nucleus, and finally inducing cell proliferation by molecules such as cyclin D1.^[122]^ In this study, the combination resulted in a better reduction of total β-catenin, and cyclin D1 levels and up-regulation of the protein expression of the tumor suppressor p53 compared to the administration of the drug alone.^[117]^

Arsenic trioxide was first widely used because of its excellent efficacy in the treatment of acute promyelocytic leukemia.^[123]^ Chaudhary^[118]^ found that compared with normal cells, arsenic trioxide combined with quercetin and aloe-emodin reduced cell viability and induced apoptosis in HepG2 calls by downregulating telomerase and antiapoptotic protein Bcl-2, and upregulating the expression of the pro-apoptotic protein Bax. It has been found that telomerase promotes programmed cell death in tumor cells; therefore, inhibition of telomerase expression may be one of the mechanisms of action of the combination of drugs against HCC.^[118]^ The inhibition of telomerase and the resulting telomere proliferation barrier in somatic cells are characteristic of the tumor suppressor pathway.^[124]^

Immune checkpoint blockade immunotherapy based on programmed cell death protein 1 (PD-1) has gained widespread attention for HCC treatment.^[125]^ Several studies have shown that combination therapies based on quercetin or PD-1 inhibitors can effectively limit HCC progression.^[126,127]^ Wu et al^[119]^ suggested that the use of PD-1 blockers to modulate tumor-associated macrophages within the TME, remove T-cell inhibitory signals, and restore the antitumor immune response within the TME provides a promising approach for HCC treatment. Subsequently, quercetin/anti-PD-1 antibody combination therapy reshaped the liver cancer TME in mice while modulating the GM and macrophage immunity. Studies have increasingly demonstrated that GM can affect the occurrence and progression of HCC by regulating the gut–liver axis.^[128]^

## 6. Nanocarrier systems of quercetin in liver cancer

Although quercetin shows good anticancer potential, its efficacy is greatly reduced due to its poor oral bioavailability, short half-life, and poor water solubility. In recent years, nanoparticles have become a popular method for the targeted delivery of drugs for the treatment of various diseases^[129,130]^ because of their high stability and ability to encapsulate different active ingredients.^[131]^ Nanocarrier systems can increase the solubility of poorly water-soluble antitumor phytopharmaceuticals, increase their bioavailability, reduce the dose of the drug, and decrease the frequency of administration, potentially reducing the toxicity and side effects of the drug.^[132]^ Nanocarrier systems have also been found to improve the therapeutic efficacy of quercetin.^[133]^ Table 3 summarizes the anti-liver cancer effects of various types of quercetin nano-formulation-based delivery systems.

**Table 3:** Anti-liver cancer effects of various types of quercetin nano-formulations-based delivery systems.

Type of nano formulations/ Nanoparticles (Quercetin Based)	Study model (both in vitro/in vivo)	Particle size (nanometres [nm])	Effects	Concentration	References
QCT-SPION-loaded Micelles (QCT-SPION-loaded Micelles)	HepG2.2.15	20–50 nm	↑cellular toxicity, inhibition of cell cycle and↑apoptosis	20, 40, 60, and 80 μM	Srisa-Nga et al^[134]^
Biodegradable QE-LiposAu nanoparticles	Huh-7	140 nm	↓Hsp70 and ↑apoptosis	20 μg/mL	Pradhan et al^[135]^
Fe_2_O_3_/Starch/Polyvinyl alcohol nanocarrier (Fe2O3/S/PVA NC)	HepG2	240–340 nm	↓cell viability and ↑apoptosis	5 μg/mL	Asl et al^[136]^

Srisa-Nga et al^[134]^ prepared a promising quercetin nanomagnetic drug delivery system by co-delivering quercetin and superparamagnetic iron oxide nanoparticles into mPEG750-b-OCL-Bz micelles. This drug delivery platform can improve drug targeting in HepG2.2.15 cells. This could not only improve efficiency but also reduce the adverse side effects of anticancer compounds and visualizers, thus improving disease monitoring and therapeutic efficacy. This system is suitable for further studies on in vivo MRI imaging and drug delivery targeting.

Pradhan et al^[135]^ developed biodegradable plasma nanoparticles loaded with quercetin and synthesized QE-loaded gold-coated liposome (QE-LiposAu) nanoparticles. QE-LiposAu nanoparticles showed increased photothermal cytotoxicity compared to LiposAu nanoparticles in Huh-7 cells, with the apoptosis rate of the former being significantly higher than that of the latter. Further, QE-LiposAu nanoparticles specifically inhibited the expression of heat shock protein 70 and induced apoptosis in HCC cells after photothermal therapy. A related study showed that the inhibitory effect of quercetin on heat shock protein 70 expression contributes to apoptosis.^[137]^

Fe_2_O_3_ has been used as a carrier for pharmaceuticals and anticancer drugs owing to its unique physicochemical and superparamagnetic properties and small size.^[138]^ In a recent study, quercetin was encapsulated in a synthesized Fe2O3/Starch/Polyvinyl alcohol nanocarrier (Fe2O3/S/PVA NC). The results showed that quercetin-loaded NC reduced HepG2 cell viability and promoted apoptosis.^[136]^

## 7. Study of quercetin-containing Traditional Chinese Medicine (TCM) against HCC

Chinese herbal medicine is a kind of TCM treatment method. As an auxiliary treatment, TCM can reduce adverse reactions, improve curative effects, and prolong patient survival time of patients.^[139]^ Many active components of TCM can promote the generation of tumor immune cells and the occurrence of antitumor reactions.^[140,141]^ TCM has a variety of antitumor effects in treating liver cancer and can play a greater role in other treatments.^[142]^ Wang identified the effective components and related genes of TCM injections in the treatment of liver cancer and determined that quercetin is an important small molecule in TCMs.^[143]^ Quercetin, a hot monomer in current research, also plays an important role in many other TCMs and formulas for treating HCC. Table 4 concludes the quercetin-containing TCM against HCC.

**Table 4:** Possible mechanisms, real modules, targets, doses, and reference of quercetin-containing Traditional Chinese Medicines against liver cancer.

TCM	Real modules (animal/ cell)	Possible mechanisms	Targets	Doses	References
*Lobelia chinensis Lour*	Hep G2, Hepa1-6;C57BL/6 mice	Proliferation, migration, and apoptosis	PTEN, AKT	50 and 100 μg/mL, 100 and 150 μg/mL; 400 mg/kg/d	Luo et al^[144]^
*Scutellaria barbata*	HepG2, Hep3B	Proliferation, migration, and apoptosis	PI3K, Akt	3.125–400 uM	Yang et al^[145]^
*Quercus mongolica Fisch*	SMMC-7721	Apoptosis, proliferation	Blocking their cell cycles	5 mg/mL	Wang et al^[146]^
*Ecliptae herba*	HepG2, Huh-7	Apoptosis, proliferation	PI3K, AKT, HIF-1A	0.15 g/mL	Pan et al^[147]^
*Radix Bupleuri*	HepG2	Mitochondria membrane potential collapse	Bax, p53, p21	100, 200 μg/mL	Kang et al ^[148]^
*Cyperus rotundus*	HepG2	Apoptosis		0.39–100 μg/mL	Mannarreddy et al^[149]^
Xihuang Pill	H22, HepG2; BALB/c mice	Proliferation, invasion, migration, autophagy	MMP-2, MMP-9, GM-CSF, G-CSF, LC3, p62, TNF-α, IL-6, IL-17A, and NF-κB	0, 25 μM, 50 μM, 100 Μm; 25 mg/kg, 50 mg/kg, 100 mg/kg	Wu et al^[150]^
Yiqi Jianpi Jiedu formula	Hep3B, HepG2	Proliferation, migration, invasion, apoptosis	MAPK3, RHOA, β-catenin, and PI3K/Akt	0 mg/mL, 1 mg/mL, 2 mg/mL, 3 mg/mL	Wu et al^[151]^

Bax = Bcl-2-associated X protein, IL-6 = interleukin-6, NF-κB = nuclear factor kappa B, TNF-α = tumor necrosis factor-alpha.

Luo^[144]^found quercetin to be an important component when studying the antitumor activity of *Lobelia chinensis Lour*. Lobelia chinensis Lour may inhibit HCC progression by inhibiting cell migration and promoting apoptosis through the PTEN/AKT signaling pathway. Yang^[145]^revealed that the mechanism of *Scutellaria barbata* treatment of HCC may be that its active components inhibit the expression of core genes and block the PI3K-AKT signaling pathway, thereby inhibiting the proliferation and migration of cancer cells and inducing cell apoptosis. Quercetin is one of the active components. Wang^[146]^ studied the antitumor effect of *Quercus mongolica Fisch* and found that quercetin, one of its bioactive components, improved the apoptosis rate and inhibited the proliferation of SMMC-7721 human liver cancer cells by inducing G0/G1 phase and G2/M phase block. A large amount of TCM evidence supports the therapeutic value of the Chinese medicine *Yinchen* in HCC. Studies have found that *Yinchen* can affect HCC by inhibiting BIRC5-targeted immune checkpoints (CTLA4 and LAG3) to activate immune cells. The results of molecular docking further verified that quercetin, the active ingredient of *Yinchen*, has good binding activity when interacting with BIRC5.^[152]^ Pan^[147]^ found that the active ingredients quercetin and wedelolactone of *Ecliptae herba* inhibited the PI3K-AKT signaling pathway, thereby inhibiting the proliferation of HCC cells and promoting HCC cell apoptosis. *Radix Bupleuri* is a TCM used for soothing the liver and promoting qi, which is considered to have hepatoprotective activity in modern pharmacological studies.^[153,154]^ Su^[148]^ showed that the water extract of *Radix Bupleuri* decreased the viability of HepG2 cells, and bupleurum enhanced the pharmacological effect of 5-fluorouracil-induced HepG2 cell death. Mannarreddy^[149]^ showed that the methanol extract of *Cyperus rotundus* showed significant anticancer activity against various cancer cell lines, including HepG2, without inhibiting non-cancer cells. Qing^[155]^ analyzed the systematic pharmacology database of TCM (TCMSP) and found that the *Radix Bupleuri–C rotundus* herb pair had a synergistic anti-HCC effect on quercetin, stigmasterol, isorhamnetin, and kaempferol.

When Wu^[150]^studied the key components of Xihuang Pill anti-HCC activity and they found that quercetin may regulate macrophage polarization through the NF-κB pathway and promote autophagy. Studies on the anti-liver cancer activity and molecular mechanism of the Yiqi Jianpi Jiedu formula have found that it mainly inhibits the proliferation, migration, and invasion of liver cancer cells through a variety of bioactive components, such as quercetin, multiple pathways, and targets defined by the PI3K/Akt pathway, β-catenin, MAPK3, and RHOA. It also promotes the apoptosis of liver cancer cells and other anticancer effects.^[151]^ SiNiSan is a prescription for the treatment of liver and spleen deficiency syndromes. Studies^[156]^ using interaction networks and molecular docking have found that SiNiSan has the unique advantages of multi-component and multi-target treatments for HCC. Quercetin is the main component that plays an anti-HCC role and acts on multiple targets in the P53 pathway. Ouyang^[157]^ found that quercetin was one of the 6 main active ingredients when discussing the potential mechanism of action of the Wuzhuyu Decoction against HCC using network pharmacology.

## 8. Clinical trials and safety studies addressing the anti-liver cancer effect of quercetin

Although numerous in vivo and in vitro studies have demonstrated the therapeutic potential of quercetin in HCC, clinical trial data specifically targeting this disease remain scarce. Nonetheless, existing clinical studies on quercetin for other conditions may provide indirect insights. For example, a phase I clinical trial of the natural flavonoid quercetin reported a sustained reduction in serum alpha-fetoprotein and alkaline phosphatase levels in a metastatic HCC patient treated with an intravenous infusion of quercetin (60 mg/m²).^[158]^ Combined with findings from other cases, this study suggests that intravenous quercetin administration is feasible and safe. However, nephrotoxicity observed in some patients with other tumors at higher doses highlights the need to establish an optimal clinical dosage for HCC. Further case data are essential before clinical translation. Another study preliminarily explored quercetin’s effect on hepatocyte injury. A 12-week randomized, double-blind, placebo-controlled trial found that consumption of quercetin-rich onions improved alanine aminotransferase levels.^[159]^ However, the absence of comprehensive liver function assessments such as bilirubin and albumin measurements limits the conclusions, warranting additional evaluations in future research. In a 28-day phase I study,^[160]^ patients with chronic hepatitis C received oral quercetin chewable tablets. Results indicated no significant improvement in liver function, but notably, no hepatic impairment exacerbation was observed. Viral load reduction was primarily observed in the 2500 to 5000 mg dose group, suggesting dose-dependent antiviral activity and good tolerability. Despite these findings, key limitations persist across all trials: small sample sizes, incomplete evaluation protocols, short treatment durations, and lack of long-term follow-up. While these studies offer valuable preliminary insights, they do not constitute direct clinical evidence for quercetin’s efficacy in HCC.

Before quercetin progresses to clinical trials, its safety profile warrants rigorous evaluation. Literature reviews indicate potential side effects including genotoxicity observed in Salmonella typhimurium genetic toxicity test,^[161]^ though this phenomenon is not consistently replicated in mammalian in vivo studies. Animal studies further suggest that high-dose quercetin may promote tumorigenesis in estrogen-dependent cancers such as breast cancer models,^[162,163]^ but whether this risk extends to human estrogen-sensitive malignancies remains unverified. Additionally, quercetin exhibits nephrotoxic effects in rodents with preexisting kidney damage,^[164]^ implying potential risks for renal-impaired patients receiving long-term high-dose regimens. Critically, the dose-dependent renal toxicity noted in the aforementioned clinical study^[158]^ aligns with preclinical findings, emphasizing the need to individualize dosing based on baseline renal function. While no systematic clinical evidence currently confirms quercetin-induced nephropathy in humans, these preclinical signals necessitate caution. Clinicians should therefore mandate medical consultation for patients prescribed high-dose quercetin monotherapy or combination regimens, particularly those with renal comorbidities.^[165]^ To advance quercetin into clinical trials, future studies must address in terms of safety evaluation, dosage of administration, and mode of administration.

## 9. Conclusion and perspectives

Currently, surgery is the preferred treatment for liver cancer. However, owing to its insidious onset, the disease has already progressed to an advanced stage when it is found, thus losing the opportunity for surgery. Other treatment modalities, such as radiotherapy, chemotherapy, immunotherapy, and targeted therapy, have the disadvantages of poor tumor sensitivity and high treatment cost, resulting in unsatisfactory therapeutic effects in liver cancer. As shown in this review article, the results of many studies have confirmed that quercetin plays an important role in anti-liver cancer effects by regulating various mechanisms to inhibit cancer cell proliferation, invasion, and migration; inducing apoptosis; and decreasing drug resistance in liver cancer treatment. Therefore, quercetin is considered a novel potential anti-liver cancer drug. However, by searching the relevant literature, the authors found that there is still a lack of relevant clinical trials of quercetin in the treatment of HCC to observe its clinical efficacy. Thus, putting it into clinical use is a major challenge that must be overcome during the research process. Before quercetin can be used clinically, there are still many issues that need to be further explored and clarified, such as bioavailability, optimal dosage for use, and dosage form. Nanocarrier systems have become a popular choice among targeted drug delivery systems in recent years, which can improve the stability, bioavailability, and solubility of quercetin to a certain extent, but the selection of the optimal dosage form and dose still needs to be further investigated. Simultaneously, efforts are increasingly focused on developing novel drug delivery systems. However, regardless of the approach, clinical safety must remain a priority during their development and application. This necessitates determining optimal dosing regimens for each formulation to maximize anticancer efficacy while ensuring safety, which requires multidimensional systematic evaluations before clinical trials. Furthermore, combination therapies integrating quercetin with chemotherapeutic agents, immune checkpoint inhibitors, or other natural compounds represent a promising research direction. Such strategies may overcome drug resistance while achieving synergistic therapeutic effects. This review has some limitations certainly. On the one hand, this paper only summarizes the mechanism of quercetin in the treatment of liver cancer in terms of proliferation, invasion, migration, apoptosis, and the reduction of drug resistance. New mechanisms are still being explored, such as iron death^[166]^ and mitophagy,^[167]^ and will be reviewed in future studies. On the other hand, when organizing this paper, the authors found that some of the pathways exerted more than one effect; quercetin exerts its anticancer effects through multiple pathways and targets. Moreover, most of the related studies have only identified the relevant target proteins, while the specific upstream and downstream mechanisms have not been systematically investigated; therefore the key mechanisms remain to be investigated in depth in the future.

In summary, quercetin exhibits substantial therapeutic promise for HCC, particularly through nanoparticle formulations engineered from quercetin and synergistic drug combinations. By integrating strategies such as nanosystem-mediated co-delivery of quercetin with other therapeutic agents, this dual approach could significantly advance clinical trial development for quercetin-based HCC therapies. Moving forward, researchers must prioritize comprehensive mechanistic exploration and translational optimization, focusing on overcoming bioavailability limitations and defining precise dosing regimens. These efforts are essential to accelerate the clinical translation of quercetin for liver cancer treatment.

## Author contributions

**Conceptualization:** Binbin Liu.

**Data curation:** Binbin Liu.

**Investigation:** Yunfeng Yu.

**Methodology:** Kewei Sun.

**Project administration:** Kewei Sun.

**Resources:** Ruoyu Wang.

**Supervision:** Yunan Wu, Kewei Sun.

**Validation:** Yunan Wu.

**Writing – original draft:** Binbin Liu.

**Writing – review & editing:** Yunfeng Yu, Ruoyu Wang.
